# Connectivity differences between consciousness and unconsciousness in non-rapid eye movement sleep: a TMS–EEG study

**DOI:** 10.1038/s41598-019-41274-2

**Published:** 2019-03-26

**Authors:** Minji Lee, Benjamin Baird, Olivia Gosseries, Jaakko O. Nieminen, Melanie Boly, Bradley R. Postle, Giulio Tononi, Seong-Whan Lee

**Affiliations:** 1 Department of Brain and Cognitive Engineering, Korea University, Seoul, Korea; 2 Wisconsin Institute for Sleep and Consciousness, Department of Psychiatry, University of Wisconsin, Madison, USA; 3 Department of Psychology, University of Wisconsin, Madison, USA; 4 Coma Science Group, GIGA-Consciousness & Neurology Department, University and University Hospital of Liege, Liege, Belgium; 5 Department of Neuroscience and Biomedical Engineering, Aalto University School of Science, Espoo, Finland; 6 Department of Neurology, University of Wisconsin, Madison, WI USA

## Abstract

The neuronal connectivity patterns that differentiate consciousness from unconsciousness remain unclear. Previous studies have demonstrated that effective connectivity, as assessed by transcranial magnetic stimulation combined with electroencephalography (TMS–EEG), breaks down during the loss of consciousness. This study investigated changes in EEG connectivity associated with consciousness during non-rapid eye movement (NREM) sleep following parietal TMS. Compared with unconsciousness, conscious experiences during NREM sleep were associated with reduced phase-locking at low frequencies (<4 Hz). Transitivity and clustering coefficient in the delta and theta bands were also significantly lower during consciousness compared to unconsciousness, with differences in the clustering coefficient observed in scalp electrodes over parietal–occipital regions. There were no significant differences in Granger-causality patterns in frontal-to-parietal or parietal-to-frontal connectivity between reported unconsciousness and reported consciousness. Together these results suggest that alterations in spectral and spatial characteristics of network properties in posterior brain areas, in particular decreased local (segregated) connectivity at low frequencies, is a potential indicator of consciousness during sleep.

## Introduction

One prominent technique to evaluate functional integration and causality in thalamocortical circuits is to examine brain activity following transcranial magnetic stimulation (TMS). Probing brain networks with TMS has shown promise for distinguishing a patient’s level of consciousness^1^. Effective connectivity assessed by TMS perturbations has been linked to the brain’s capacity for consciousness^2^ and evidence suggests that differences between consciousness and unconsciousness can be detected by measuring the complexity of the response to TMS perturbations^3^. For example, using concurrent TMS and high-density electroencephalography (EEG), it has been found that effective connectivity breaks down during non-rapid eye movement (NREM) sleep^4–6^; general anesthesia with propofol, xenon, or midazolam^7–9^; and unresponsive wakefulness syndrome/vegetative state^6,10,11^. In contrast, complex and widespread responses to TMS are triggered during conscious states (e.g., normal wakefulness, dreaming, minimally conscious state, and ketamine anesthesia)^2,8,12,13^.

Recent studies have found that power and connectivity in the delta (1–4 Hz) frequency band increase during unconsciousness. This increase is likely attributable to the coordinated bistability of neuronal transitions between up- and down-states^14,15^. Specifically, slow-wave activity (SWA), which occurs prominently during NREM sleep and manifests as delta activity in the EEG, is caused by rapidly alternating neuronal hyperpolarized (OFF) and depolarized (ON) states^16^. These bimodal changes between neuronal down- and up-states have been referred to as bistability of thalamocortical circuits^17^, and have been suggested to be a key factor in the disintegration of brain complexity that occurs during the loss of consciousness^17^.

Previous studies have mostly compared wakefulness with sleep or anesthesia to evaluate features associated with the level of consciousness in healthy individuals^14,18,19^. However, such studies are confounded by other changes that occur across global state shifts, such as changes in the cardiovascular, respiratory, and neuromuscular systems^20^. To address this issue, recent studies have measured the presence or absence of consciousness within the same physiologically categorized state using a within-state paradigm^21–23^. For example, consciousness during NREM sleep can be assessed with serial-awakenings, in which individuals are woken up many times throughout the night to report whether they were having an experience just prior to the awakening^22^. Awakenings with reports of conscious can then be contrasted with awakenings with reports of unconsciousness within each vigilance state (i.e., rapid eye movement (REM) or NREM sleep) in this within-state design. Using this approach, a recent study found that spectral power in the delta band in posterior cortex was higher during reported unconsciousness than during reported consciousness^23^. Furthermore, using a within-state design in NREM sleep, it has been found that TMS triggers a larger negative EEG peak amplitude during reported unconsciousness than during reported consciousness, indicating that differences in consciousness within the same physiological state are related to local alterations in the cortical bistability of posterior brain regions^21^.

In this study, we built on these previous findings to investigate differences in the network topology of the brain during the presence or absence of consciousness (CE or NCE, respectively) in response to TMS perturbation. We used a serial-awakening paradigm during NREM sleep to evaluate the level of consciousness within the same physiological state. We evaluated the synchronization of oscillatory activity following TMS perturbation by quantifying the TMS-induced phase-locking value (PLV) between channels. We then employed graph theoretic analysis to study changes in brain complexity by quantifying network properties (e.g., network segregation and integration) of interacting neuronal elements^17,24^ following TMS perturbation. We hypothesized that the level of consciousness would be inversely related to the strength of phase locking at low frequencies, particularly in posterior cortical regions, due to changes in cortical bistability in thalamocortical circuits. We also predicted that the local aspects of functional segregation would occur strongly in unconsciousness.

## Results

### Spectral power in CE and NCE at 0–400 ms

We first measured TMS-induced and TMS-evoked spectral power. No significant differences between CE and NCE were observed in induced or evoked power in electrodes over either frontal or parietal regions (Table 1 and Supplementary Fig. S1).

**Table 1:** Statistical values related to the TMS-induced/evoked spectral power.

			Delta band	Theta band	Alpha band	Beta band	Gamma band
Induced power	Frontal region	*t*-value	−0.69	−0.79	−2.32	−1.13	−0.40
		*p*-value	0.56	0.50	0.09	0.39	0.82
	Parietal region	*t*-value	−0.31	−0.29	−1.28	−1.27	−0.56
		*p*-value	0.79	0.81	0.31	0.26	0.70
Evoked power	Frontal region	*t*-value	−1.38	0.36	−0.57	−0.01	1.64
		*p*-value	0.25	0.74	0.59	0.99	0.15
	Parietal region	*t*-value	−1.79	−0.91	−0.83	−0.53	1.54
		*p*-value	0.12	0.46	0.41	0.61	0.19

Note: *N* = 6; *df* = 5 for all tests.

### PLV in CE and NCE at 0–400 ms

**Figure 1:**
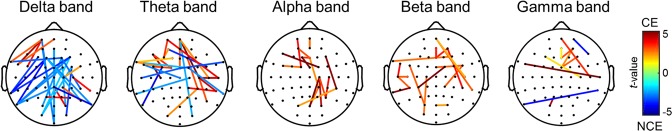
Differences in PLV-based connectivity between CE and NCE. Significant differences between CE and NCE are plotted (*p* < 0.05, FDR-corrected). The color bars indicate the *t*-value of the difference between CE and NCE. Red edges indicate connections that are stronger in CE than in NCE, whereas blue edges indicate connections that are stronger in NCE than in CE. PLV = phase-locking value; FDR = false discovery rate; CE = conscious experience; NCE = no conscious experience.

We observed significant differences in PLV-assessed connectivity in NREM sleep in response to TMS perturbation between NCE and CE. PLV was higher in NCE than in CE in the delta and theta bands (*p* < 0.05 with false-discovery-rate (FDR) correction for channels and Bonferroni correction for frequency), with widespread phase synchrony in the delta band during NCE. Higher PLV was also observed between several electrodes in the alpha and beta bands in CE compared to NCE (*p* < 0.05 with FDR correction for channels and Bonferroni correction for frequency) (Fig. 1).

### Network properties based on PLV at 0–400 ms

**Figure 2:**
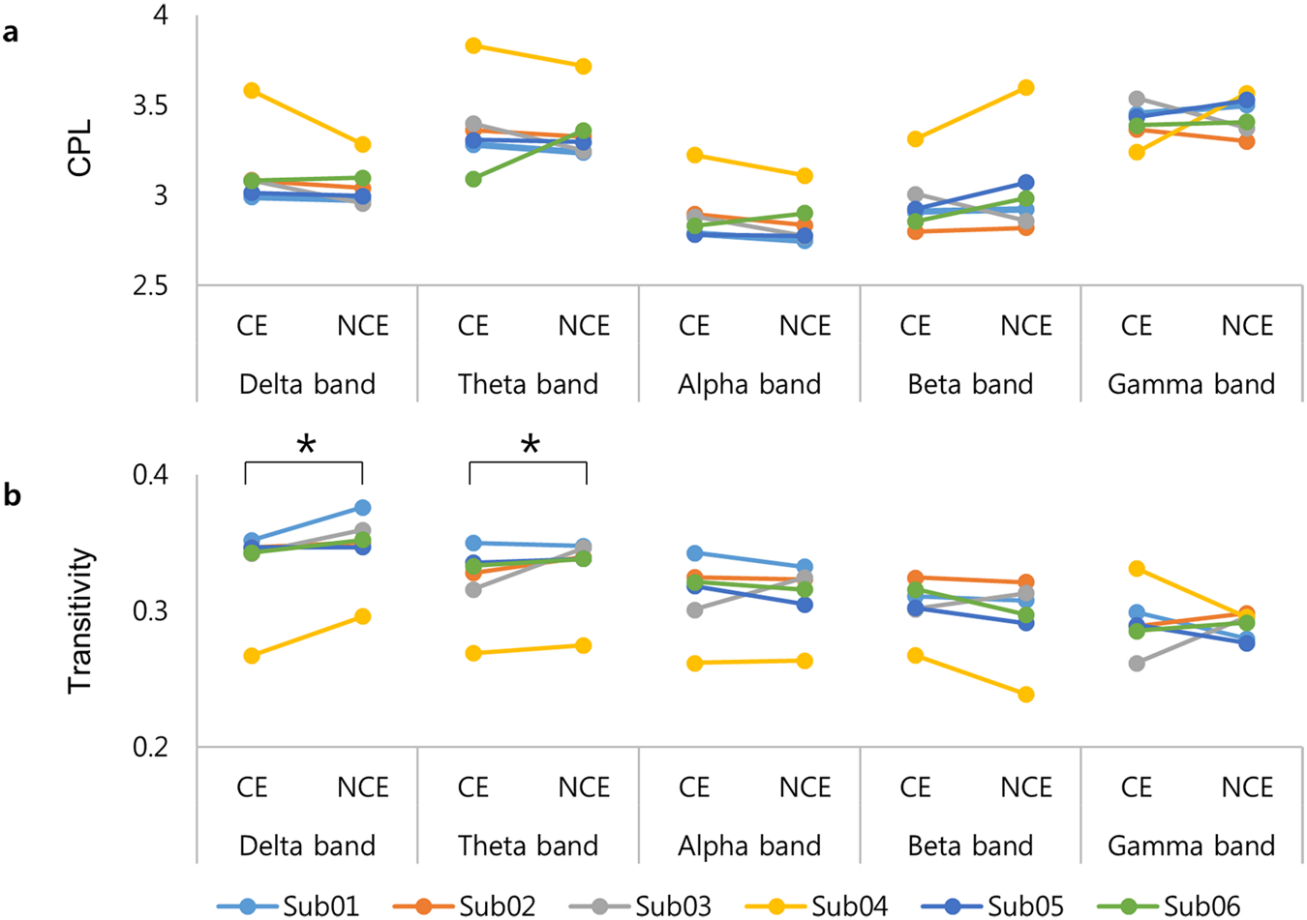
Global network properties of CE and NCE based on PLV. (**a**) Characteristic path length (CPL) and (**b**) transitivity in all frequency bands for all subjects. The error bars show the standard error. The asterisks indicate significant differences in the mean transitivity between CE and NCE using multi-threshold permutation correction. PLV = phase-locking value; CE = conscious experience; NCE = no conscious experience.

Figure 2 shows the global network properties of CE and NCE based on PLV. Transitivity was significantly higher at low frequencies in NCE compared to CE (delta, *p* = 0.027; theta, *p* = 0.026 with multi-threshold permutation correction (MTPC)^25^ for each frequency). No significant differences in transitivity were observed between CE and NCE at other frequencies (alpha, *p* = 0.46; beta, *p* = 0.90; gamma, *p* = 0.41 with MTPC for each frequency). No significant differences in the characteristic path length were found between CE and NCE (delta, *p* = 0.95; theta, *p* = 0.99; alpha, *p* = 0.93; beta, *p* = 0.43; gamma, *p* = 0.25 with MTPC for each frequency). In the delta band, transitivity was higher in NCE than in CE in 6/6 subjects. In the theta band, transitivity was higher in NCE than in CE in 5/6 subjects.

**Figure 3:**
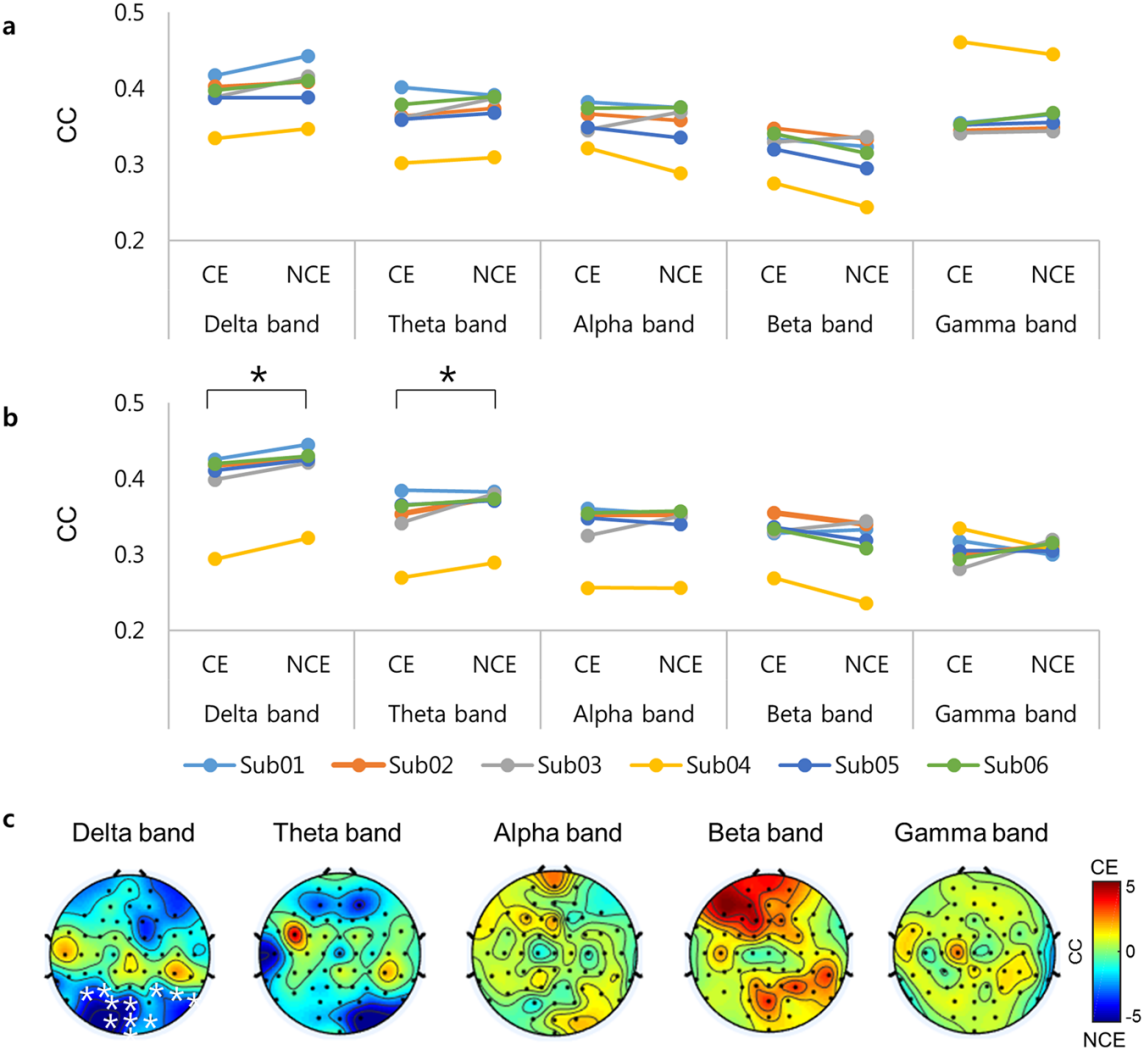
Local network properties of CE and NCE based on PLV. The clustering coefficients in the (**a**) frontal and (**b**) parietal regions in the studied frequency bands for all subjects. Black asterisks indicate significant differences in the mean clustering coefficient between CE and NCE according to MTPC. (**c**) Topographies of the differences in the local processing (clustering coefficient) between CE and NCE. The colors indicate the *t*-value of the difference between CE and NCE. Red regions indicate that the clustering coefficient is stronger in CE than in NCE, whereas blue regions indicate that the clustering coefficient is stronger in NCE than in CE. White asterisks indicate channels with significant differences between CE and NCE according to SnPM. SnPM = statistical non-parametric mapping; CC = clustering coefficient; CE = conscious experience; NCE = no conscious experience; PLV = phase-locking value.

We next examined the clustering coefficient of frontal and parietal scalp regions. The clustering coefficient was significantly higher in the parietal region in NCE than in CE at low frequencies (delta, *p* = 0.031; theta, *p* = 0.035 with MTPC for each frequency). A topographical analysis revealed that the clustering coefficient was higher in parietal–occipital regions in the delta band in NCE than in CE (*p* = 0.046, statistical non-parametric mapping [SnPM] cluster corrected; Fig. 3c). In contrast, there were no significant differences in the clustering coefficient of frontal electrodes at low frequencies (delta, *p* = 0.11; theta, *p* = 0.08 with MTPC for each frequency). Similar to the global properties, we observed no significant differences in the clustering coefficient between CE and NCE in either frontal or parietal regions at other frequencies (frontal region: alpha, *p* = 0.44; beta, *p* = 0.95; gamma, *p* = 0.40. parietal region: alpha, *p* = 0.49; beta, *p* = 0.93; gamma, *p* = 0.28 with MTPC for each frequency) (Fig. 3a and 3b). The clustering coefficient was higher in NCE than in CE in the delta band in the parietal regions in 6/6 subjects. In addition, the clustering coefficient was higher in NCE than in CE in the theta band in the parietal regions in 5/6 subjects, but an opposite pattern was observed in one subject.

### Directionality based on GC at 0–400 ms

Table 2 shows GC in frequency domains. There were no significant differences between CE and NCE in frontal-to-parietal directionality or parietal-to-frontal directionality (Table 3; top row). Additionally, we compared frontal-to-parietal with parietal-to-frontal directionality within CE and within NCE. There were no significant differences between frontal-to-parietal directionality and parietal-to-frontal directionality in CE or in NCE (Table 3; bottom row)

**Table 2:** Directionality in CE and NCE based on Granger causality.

	Delta band		Theta band		Alpha band		Beta band		Gamma band	
	CE	NCE	CE	NCE	CE	NCE	CE	NCE	CE	NCE
GC_f→p_	0.145 ± 0.096	0.247 ± 0.277	0.127 ± 0.085	0.178 ± 0.189	0.074 ± 0.066	0.195 ± 0.226	0.038 ± 0.026	0.133 ± 0.150	0.035 ± 0.026	0.110 ± 0.144
GC_p→f_	0.165 ± 0.156	0.161 ± 0.179	0.128 ± 0.101	0.170 ± 0.191	0.086 ± 0.058	0.206 ± 0.231	0.056 ± 0.038	0.172 ± 0.178	0.045 ± 0.038	0.158 ± 0.184

Frontal-to-parietal GC connectivity and parietal-to-frontal GC connectivity in all frequency bands are described. Data are presented as mean Granger causality ± standard deviation. GC_f→p_ = frontal-to-parietal directionality of Granger causality; GC_p→f_ = parietal-to-frontal directionality of Granger causality; CE = conscious experience; NCE = no conscious experience.

**Table 3:** Statistical values related to the directionality based on Granger causality.

			Delta band	Theta band	Alpha band	Beta band	Gamma band
CE vs. NCE	GC_f→p_	*t*-value	−1.12	−0.60	−1.16	−1.40	−1.44
		*p*-value	0.44	0.58	0.37	0.25	0.23
	GC_p→f_	*t*-value	0.04	−0.42	−1.09	−1.39	−1.33
		*p*-value	0.92	0.68	0.41	0.28	0.22
GC_f→p_ vs. GC_p→f_	CE	*t*-value	−0.34	−0.04	−0.61	−2.15	−0.47
		*p*-value	0.78	0.99	0.72	0.09	0.69
	NCE	*t*-value	1.53	0.33	−0.32	−1.86	−2.22
		*p*-value	0.13	0.83	0.82	0.12	0.10

Note: *N* = 6; *df = *5 for all tests.

GC_f→p_ = frontal-to-parietal directionality of Granger causality; GC_p→f_ = parietal-to-frontal directionality of Granger causality; CE = conscious experience; NCE = no conscious experience.

### Network properties and directionality at 600–1000 ms

We next analyzed the EEG minimally affected by TMS (at 600–1000 ms after TMS). There were no significant differences between CE and NCE in the PLV-based characteristic path length or transitivity (global network properties) in any of the studied frequency bands (characteristic path length: delta, *p* = 0.88; theta, *p* = 0.90; alpha, *p* = 0.83; beta, *p* = 0.83; gamma, *p* = 0.36. transitivity: delta, *p* = 0.18; theta, *p* = 0.23; alpha, *p* = 0.36; beta, *p* = 0.37; gamma, *p* = 0.36) (Supplementary Fig. S2). In addition, regarding the local network properties, there were no significant differences between CE and NCE in clustering coefficient of the network recorded at electrodes over the frontal (delta, *p* = 0.46; theta, *p* = 0.29; alpha, *p* = 0.32; beta, *p* = 0.56; gamma, *p* = 0.75) or parietal (delta, *p* = 0.16; theta, *p* = 0.39; alpha, *p* = 0.36; beta, *p* = 0.43; gamma, *p* = 0.81) regions (Supplementary Fig. S3).

We investigated the directionality minimally affected by TMS (Supplementary Table S1). There was no significant difference between CE and NCE in frontal-to-parietal directionality or parietal-to-frontal directionality (Supplementary Table S2; top row). We also compared frontal-to-parietal directionality and parietal-to-frontal directionality within the same state. No significant differences were observed in any frequency band in CE or NCE (Supplementary Table S2; bottom row). Additionally, there was a difference between front-to-back and back-to-front within NCE in the delta band (*p* = 0.032); however, after multiple comparisons, this difference was not significant.

## Discussion

We analyzed changes in brain connectivity and network topology associated with the level of consciousness during TMS perturbation in NREM sleep. The analysis considered within-state differences in EEG during NREM sleep to eliminate confounding factors caused by physiological differences across global state shifts, such as those that occur between sleep and wake. Our data show that compared to reports of consciousness, reports of unconsciousness were associated with increased phase-locking at low frequencies (<4 Hz) as well as increased segregated, localized neuronal processing (as measured by the clustering coefficient and transitivity) in posterior brain regions at low frequencies. However, no significant difference of directed connectivity between CE and NCE was observed in any frequency band.

At the network level, cortical bistability interferes with the connections among distributed brain regions for recurrent processing^26^ and brain complexity^1,23^. Therefore, increased phase-locking at low frequencies during unconsciousness may reflect coordinated bistability that prevents adequate integration of brain areas important for consciousness^14^. Indeed, the breakdown of TMS-induced effective connectivity during unconscious states^4,27^ has been suggested to be a consequence of neuronal bistability, which disrupts cortico-cortical interactions during unconsciousness. Consistent with this, our analysis revealed strong phase-locking at low frequencies during reported unconsciousness. This result is consistent with other studies that have investigated loss of consciousness in other contexts. For example, low-frequency connectivity has been found to be stronger in patients in minimally conscious state and with unresponsive wakefulness syndrome than in healthy individuals, whereas high-frequency connectivity appears stronger in healthy individuals than in these patients^28^. Moreover, connectivity in the delta band increases but connectivity in the alpha band decreases during propofol- and midazolam-induced unconsciousness compared to wakefulness^14,29,30^. The present study demonstrated that PLV increased at the alpha and beta bands in CE compared to NCE. These findings further suggest that changes in high-frequency connectivity are an indicator of the disintegration of dynamic connectivity during the loss of consciousness.

The current study showed that at low frequencies the clustering coefficient of the network recorded over parietal and occipital areas was higher in NCE than in CE. Because high clustering indicates that local information efficiency is high in these regions^31^, these findings support the view that the posterior cortex plays a role in consciousness. In addition, information flow among distributed cortical regions, related to consciousness, is disrupted during unconsciousness due to bistability^21^. These results are consistent with the findings in other studies, which have identified a “posterior hot zone” in the brain as a reliable neuronal correlate of consciousness across multiple behavioral states^32–36^. According to our results, differences in the clustering coefficient in low-frequency oscillations in response to TMS perturbation between the presence and the absence of consciousness occur primarily in a parietal–occipital region during NREM sleep.

Importantly, Siclari *et al*.^23^ found that low-frequency activity is reduced in the posterior hot zone during dreaming compared with unconsciousness in both REM and NREM sleep. In other words, the absence of conscious experiences is associated with increased low-frequency activity in the posterior hot zone during sleep. Recent studies also indicate that brain connectivity increases at low frequencies during anesthetic-induced unconsciousness in parietal but not frontal regions^14,30^. These findings are consistent with the view that changes in local circuits in the posterior hot zone (and particularly in low-frequency activity) are associated with the level of consciousness^34^.

The current study also investigated Granger causality (GC)-based directionality. No significant differences in GC-based brain connectivity were observed between CE and NCE. Previous studies have found mixed results regarding the relationship between consciousness and GC. Some studies have shown that GC during general anesthesia is higher compared to wakefulness^37^, whereas other studies have reported that GC in anesthesia is reduced compared to wakefulness^38^. In terms of directionality, some studies have observed differences between consciousness and unconsciousness in frontal-to-parietal directional connectivity^37,39^, whereas other studies have observed differences in the parietal-to-frontal direction^38^. The unidirectional interaction represented by GC is related to mechanisms of information flow in cortical circuits in terms of the anatomical connectivity principle of reciprocity in the cortex^37^. However, further research is needed on the relationship between causal directionality and consciousness.

We found no significant differences in TMS-induced/evoked spectral power between CE and NCE. The findings do not contradict our phase-synchronization results because phase synchronization, which represents the connectivity of the spatial oscillatory system in the cortex, is independent of the amplitude of the signals^40,41^. As mentioned earlier, slow wave activity (SWA) is an indicator of NREM sleep, and it can be used to distinguish NREM sleep from wakefulness^42^. However, differences in SWA power are not always observed between consciousness and unconsciousness. For example, no changes in SWA were observed during sevoflurane-induced unconsciousness^43^. It is possible that network measures of coordinated bistability and segregation capture the mechanism of unconsciousness more accurately than low-frequency power; therefore, further studies are needed to compare the predictive power of these different measures across different domains. Collectively, the current results indicate that network measures can provide useful markers of consciousness, but future work is needed to test whether these measures are more sensitive than SWA in distinguishing the level of consciousness.

The current study has several limitations. First, phase-locking and GC are sensitive to the effects of volume conduction when applied to EEG data in channel space^44,45^. To learn more about the underlying cortical networks, further studies should also apply other connectivity estimators, such as the phase lag index, which is insensitive to volume conduction or analyze data in source space. Second, in future studies, it could be useful to include other measures of directionality, such as partial directed coherence, which explains the direction of flow as a frequency-domain GC measure^46^.

In conclusion, we investigated TMS-induced synchronization and directionality related to sleep consciousness using graph theoretical analysis on TMS–EEG data. Our results indicate that alterations in spectral and spatial characteristics of network properties in posterior brain areas are associated with changes in the level of consciousness. Altogether, the data suggest that connectivity in parietal–occipital regions, particularly decreased local (segregated) connectivity at low frequencies in these areas, is a potential indicator of consciousness.

## Methods

### Data acquisition and pre-processing

The dataset analyzed here, along with a detailed description of the experimental protocol, has been published in a previous study by Nieminen *et al*.^21^. Here, we describe the main experimental details.

Six healthy subjects (5 males and 1 female; age 23.7 ± 3.2 years [mean ± standard deviation]) were included in the study. The study was approved by the University of Wisconsin–Madison Institutional Review Board and was carried out in accordance with the Declaration of Helsinki. All methods were performed in accordance with the relevant guidelines and regulations. All participants provided signed informed consent.

Subjects engaged in a serial-awakening paradigm^22^ (four to five nights per participant). EEG signals were recorded using a 60-channel TMS-compatible EEG amplifier (Nexstim eXimia, Nexstim Plc, Finland) according to the international 10–10 system. The sampling rate was 1450 Hz. After at least three minutes of NREM sleep, single-pulse TMS was delivered to the medial superior parietal cortex using a figure-of-eight coil (Focal Bipulse, Nexstim Plc, Finland) at random interstimulus intervals (2–2.3 seconds). To avoid auditory-evoked responses due to the TMS application, noise masking was applied through earphones and a thin foam pad was placed between the scalp and the coil. We used a neuronavigation system (eXimia NBS, Nexstim Plc, Finland) to perform accurate and reproducible stimulation of the cortical target point indicated on the T1 magnetic resonance images of the subject’s head in all night sessions. The maximum electric field at the TMS target was between 100 and 130 V/m (70–83% of the maximum stimulator output). After each TMS sequence, participants were awoken from NREM sleep by an alarm sound lasting 1.5 seconds and were asked whether they were experiencing anything before the alarm. The awakenings were divided into two categories based on the subjective reports: 1) conscious experience (CE, with or without the recall of the content of the experience) and 2) no conscious experience (NCE). CE and NCE accounted for 58.3% and 41.7% of the NREM-sleep awakenings, respectively.

Data were pre-processed with Matlab (The MathWorks, Inc., MA, USA). The sleep scoring of the recorded EEG data was performed according to standard AASM criteria^47^. Trials with artifacts or brief arousals were manually removed using The SiSyPhus Project Matlab program (University of Milan, Italy). The EEG data in the first 15 ms after TMS were rejected and linearly interpolated to avoid disturbance from TMS-associated artifacts. The data were band-pass filtered between 1.5 and 45 Hz (a second-order Butterworth filter applied in the both forward and backward direction) and down-sampled to 362.5 Hz. The signals of −400–1000 ms were epoched around the TMS pulses and baseline-corrected using a 400-ms-long baseline interval. The baseline-corrected changes within a short time period (0–400 ms) after the TMS pulse were used as the stimulation-evoked state. The reason for choosing this time period was that TMS-evoked potentials in this time period significantly differed between CE and NCE in a previous study^21^. In addition, the brain’s early response to TMS-induced perturbation were used for measuring the level of consciousness^1^ and difference in TMS-induced potentials due to changes in parameters of TMS were found in the early response^48^. We analyzed also a time period (600–1000 ms) less affected by TMS pulses. We assumed that the signals of 600–1000 ms after TMS were minimally affected by the stimulation to investigate the ‘resting-state EEG’. Bad channels of each dataset were visually detected and interpolated. The data were re-referenced to the average of all electrode potentials.

The pre-processed TMS–EEG data in the last 30 seconds before each awakening were extracted for further analysis because this time interval has been shown to be suitable to distinguish between CE and NCE during NREM sleep^21^. A maximum of 14 trials per session was used to explore the TMS-evoked EEG response. EEG signals were divided into five frequency bands using finite impulse response filters: delta (1.5–4 Hz), theta (4–8 Hz), alpha (8–13 Hz), beta (13–30 Hz), and gamma (30–40 Hz) bands. To explore network properties in specific regions, two groups of channels were identified: frontal region (13 channels: Fp1–2, Fpz, AF1–2, AFz, F1–2, F5–8, and Fz) and parietal region (7 channels: PO3–4, POz, O1–2, Oz, and lz).

### Spectral power analysis

To assess TMS-evoked spectral power, we calculated power spectral density using the EEGLAB toolbox^49^ separately for the CE and NCE. In each channel, single trials were time–frequency decomposed using Fast Fourier Transform with power baseline (−400–0 ms). For TMS-induced power, transformation of EEG signals was performed for each trial and then averaged. For TMS-evoked power, time–frequency decomposition was performed on the averaged trials. The spectral power over all channels in frontal and parietal regions was averaged in each frequency band.

### Connectivity estimation

PLV^50^ for all channel pairs (60 × 60 pairs) was used as a connectivity estimator for averaged trials. The PLV at time *t* is defined as follows:

1 $${{\rm{PLV}}}_{t,i,k}=\frac{1}{N}|{\sum }_{n=1}^{N}\exp (j{\theta }_{i,k}(t,n))|$$

where ${\theta }_{i,k}(t,n)={\varphi }_{i}(t,n)-{\varphi }_{k}(t,n)$, and ${\theta }_{i,k}(t,n)$ is the phase difference between channels *i* and *k* in trial *n*; *N* is the number of trials. Instantaneous phase was extracted using Hilbert transform. After calculating the phase difference, the PLV was obtained by dividing the number of trials. PLV_*t*,*i*,*k*_ thus indicates the inter-trial variability of the phase difference at time *t* between two channels^51^.

The 60 × 60 subject-wise, band-wise PLV matrix was calculated. PLV indicates the transient synchrony based on the instantaneous phase difference between two channels^52^. This connectivity matrix was thresholded to maintain only the strongest links and repress false positive connections. The connection strength between two channels was maintained above the threshold density, whereas the remaining connections were set to zero. We used multi-thresholded density to identify sustained significant effects. The weighted connectivity estimators from 20% (sparse connection) to 90% (abundant connection) of maximum connection density were constructed with 10% steps^53^.

### Network properties

The brain network consists of nodes (EEG channels) and edges (the weighted connectivity between two EEG channels). Based on PLV, we calculated global network properties (characteristic path length and transitivity) in all channels and local network properties (clustering coefficient) in frontal and parietal regions in each frequency band using the Brain Connectivity Toolbox^54^. Characteristic path length suggests the functional integration, whereas transitivity suggests the functional segregation in the brain network^54^. The characteristic path length refers to the average shortest path length between all pairs of nodes in the brain network^55^. The transitivity is the number of triangles in the network as segregation of the network in all pairs of nodes, but not in an individual node^56^. Characteristic path length and transitivity in the network are the most generally used indicators of functional integration and segregation, respectively^54^. Similarly, the clustering coefficient locally is the fraction of triangles around each individual node as measures of segregation^55^. In summary, the characteristic path length indicates the integration in the brain connectivity, whereas the transitivity and clustering coefficient indicate the segregation in the cortical network.

### Directionality based on Granger causality (GC)

We calculated GC in the aforementioned five frequency bands using the MVGC toolbox^57^ based on advanced VAR (vector autoregressive) model theory. Akaike information criterion^58^ was used for estimating appropriate model order to maximize auto-covariance lags. EEG signals were then transformed to auto-covariance data based on MVGC routines. Subsequently, pairwise spectral GC was conducted in the observed auto-covariance sequences.

### Statistical analysis

A non-parametric permutation test was used for comparing the TMS-induced and evoked spectral power between CE and NCE.

To compare the PLV between CE and NCE in NREM sleep, a non-parametric permutation test was performed (*r* = 10,000). FDR correction was also performed across channels separately for each frequency band. Then, Bonferroni correction was performed for all frequency bands. We compared the network properties (global and local properties) between CE and NCE using MTPC^25^. MTPC computes statistical mapping on graph properties across multiple thresholds and verifies significant effects using thresholds based on the cluster-enhanced permutation correction approach. The network properties were calculated by applying multiple thresholds. Mann–Whitney U test on the network properties was used between the correct group assignments (conscious experience vs. no conscious experience) for each threshold. Then, the group assignments were permuted based on the null distribution at each threshold, and the statistics for each threshold and permutation were recalculated. The maximum statistics were taken across all thresholds for each permutation. The critical value was identified from the null test statistics. We set the confidence level to 5% and the number of permutations to 10,000.

For local (electrode-specific/topographical) properties, we applied SnPM^59^, a cluster-based correction for multiple comparisons, to compare the spatial characteristic in the topography between CE and NCE at the thresholded density corresponding to the maximum *t*-value (*r* = 10,000). For directionality based on GC, we performed the non-parametric permutation test with Bonferroni correction to investigate the differences between CE and NCE. For all statistical tests, the significance level was set at α = 0.05.

## Supplementary information

Supplementary information

## Data Availability

All data analyzed during this study are included in published article^21^, and are available on reasonable request.
