# Supplementary material for: Connectivity differences between consciousness and unconsciousness in non-rapid eye movement sleep: a TMS–EEG study
Source: Sci Rep. 2019 Mar 26;9:5175. doi: 10.1038/s41598-019-41274-2 (PMC6435892; doi:10.1038/s41598-019-41274-2)
Supplement: Supplementary file 1 — Supplementary information [file 41598_2019_41274_MOESM1_ESM.docx]

**Supplementary information**

**Connectivity differences between consciousness and unconsciousness in non-rapid eye movement sleep: a TMS–EEG study**

Minji Lee^1^, Benjamin Baird^2^, Olivia Gosseries^2,3,4^, Jaakko O. Nieminen^2,5^, Melanie Boly^2,6^, Bradley R. Postle^2,3^, Giulio Tononi^2^, and Seong-Whan Lee^1*^

^1^Department of Brain and Cognitive Engineering, Korea University, Seoul, Korea

^2^Wisconsin Institute for Sleep and Consciousness, Department of Psychiatry, University of Wisconsin, Madison, USA

^3^Department of Psychology, University of Wisconsin, Madison, USA

^4^Coma Science Group, GIGA-Consciousness & Neurology Department, University and University Hospital of Liege, Liege, Belgium

^5^Department of Neuroscience and Biomedical Engineering, Aalto University School of Science, Espoo, Finland

^6^Department of Neurology, University of Wisconsin, Madison, WI, USA

* Corresponding author:

Seong-Whan Lee

Department of Brain and Cognitive Engineering, Korea University

145 Anam-ro, Seongbuk-gu, Seoul 02841, Korea

E-mail: sw.lee@korea.ac.kr

**Supplementary figures**


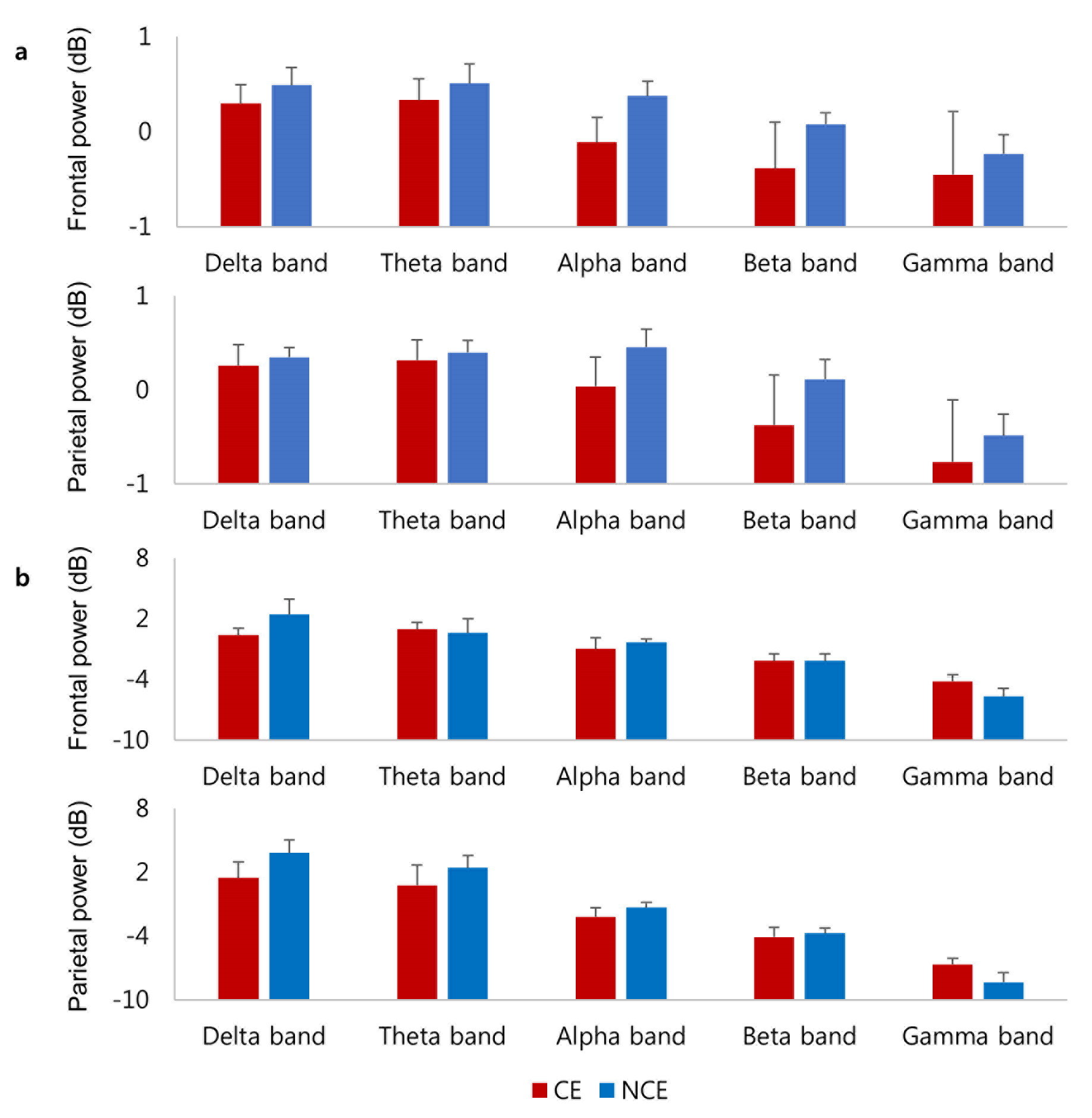


**Supplementary Figure S1. Spectral power of CE and NCE.** Mean (a) TMS-induced power and (b) TMS-evoked power recorded at scalp electrodes over the frontal and parietal regions in the studied frequency bands. The error bars show the standard error. There were no significant differences in the TMS-induced or TMS-evoked power between CE and NCE. TMS, transcranial magnetic stimulation; CE = conscious experience; NCE = no conscious experience.


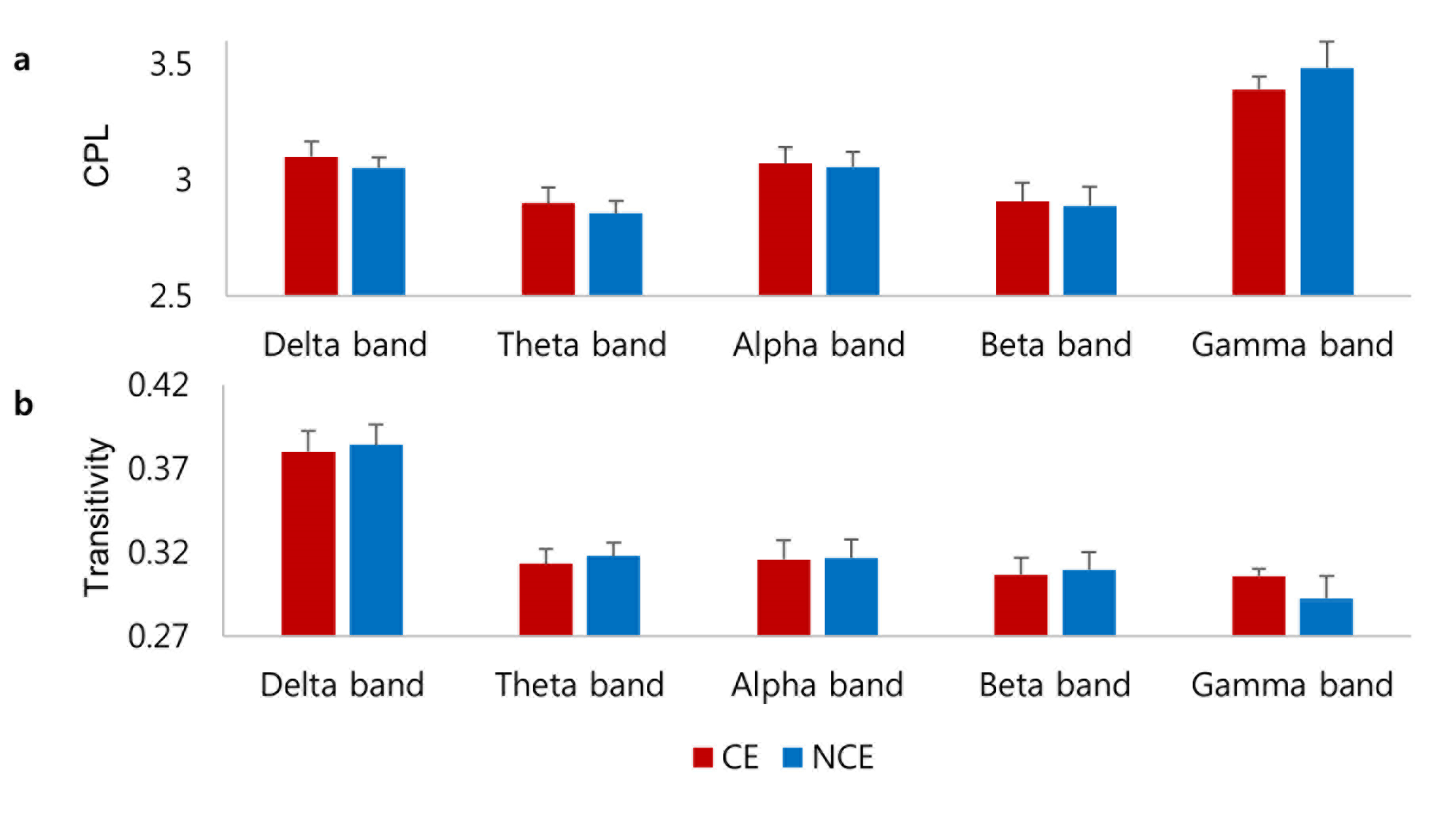


**Supplementary Figure S2. Global network properties of CE and NCE based on the EEG data minimally affected by TMS (at 600–1000 ms after TMS).** (a) Mean CPL and (b) mean transitivity in all frequency bands. The error bars show the standard error. CPL = characteristic path length, CE = conscious experience; NCE = no conscious experience, TMS = transcranial magnetic stimulation.


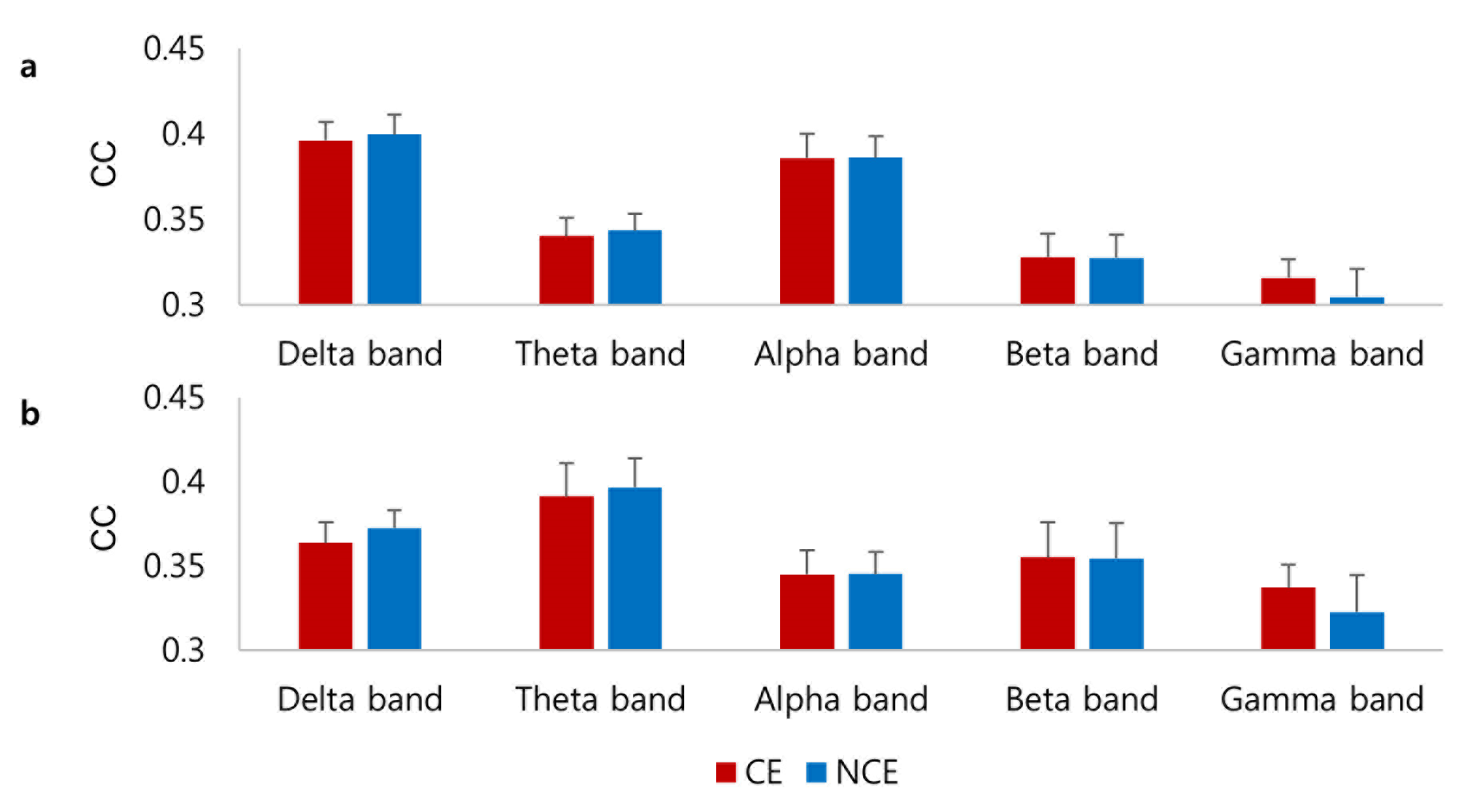


**Supplementary Figure S3. Local network properties of CE and NCE based on PLV on the EEG data minimally affected by TMS (at 600–1000 ms after TMS).** The mean clustering coefficient in the (a) frontal and (b) parietal electrode regions in all studied frequency bands. The error bars show the standard error. CC = clustering coefficient; CE = conscious experience; NCE = no conscious experience; TMS = transcranial magnetic stimulation.

**Supplementary Table S1. Directionality between CE and NCE based on Granger causality on the EEG data minimally affected by TMS (at 600–1000 ms after TMS).** Frontal-to-parietal causal connectivity and parietal-to-frontal causal connectivity in all frequency bands are described. The data are presented as mean Granger causality ± standard deviation. * indicates a significant difference (*p* < 0.05). GC_f→p_ = frontal-to-parietal directionality of Granger causality; GC_p→f_ = parietal-to-frontal directionality of Granger causality; CE = conscious experience; NCE = no conscious experience.

| Frequency | Delta band | | Theta band | | Alpha band | | Beta band | | Gamma band | |
| --- | --- | --- | --- | --- | --- | --- | --- | --- | --- | --- |
| Condition | CE | NCE | CE | NCE | CE | NCE | CE | NCE | CE | NCE |
| GC_f→p_ | 0.184  ± 0.166 | *0.166  ± 0.223 | 0.159  ± 0.107 | 0.151  ± 0.213 | 0.090  ± 0.078 | 0.148  ± 0.211 | 0.048  ± 0.052 | 0.12  ± 0.160 | 0.059  ± 0.058 | 0.063  ± 0.063 |
| GC_p→f_ | 0.188  ± 0.203 | *0.100  ± 0.158 | 0.167  ± 0.118 | 0.112  ± 0.180 | 0.103  ± 0.051 | 0.126  ± 0.216 | 0.065  ± 0.064 | 0.113  ± 0.195 | 0.079  ± 0.065 | 0.068  ± 0.080 |

**Supplementary Table S2. Statistical value in the directionality based on Granger causality on the EEG data minimally affected by TMS (at 600–1000 ms after TMS).** GC_f→p_ = frontal-to-parietal directionality of Granger causality; GC_p→f_ = parietal-to-frontal directionality of Granger causality; CE = conscious experience; NCE = no conscious experience.

|  |  |  | Delta band | Theta  band | Alpha  band | Beta  band | Gamma  band |
| --- | --- | --- | --- | --- | --- | --- | --- |
| CE  vs.  NCE | GC_f→p_ | *t*-value | 0.34 | 0.09 | −0.58 | −0.88 | −0.09 |
|  |  | *p*-value | 0.75 | 0.95 | 0.72 | 0.53 | 0.98 |
|  | GC_p→f_ | *t*-value | 1.90 | 0.89 | −0.25 | −0.55 | 0.21 |
|  |  | *p*-value | 0.16 | 0.34 | 0.94 | 0.74 | 0.87 |
| GC_f→p_  vs.  GC_p→f_ | CE | *t*-value | −0.06 | −0.23 | −0.56 | −1.30 | −0.56 |
|  |  | *p*-value | 0.98 | 0.87 | 0.64 | 0.28 | 0.56 |
|  | NCE | *t*-value | 2.27 | 2.01 | 1.63 | −0.03 | −0.38 |
|  |  | *p*-value | 0.03 | 0.12 | 0.15 | 0.99 | 0.65 |

Note: *N* = 6; *df* = 5 for all tests.
